# The Dynamics of Neuroinflammation in Traumatic Brain Injury: Molecular Markers Useful for Establishing the Post-Traumatic Interval in Forensic Practice

**DOI:** 10.3390/ijms27042049

**Published:** 2026-02-22

**Authors:** Sorin Hostiuc, Mugurel-Constantin Rusu

**Affiliations:** 1 Department of Legal Medicine and Bioethics, Carol Davila University of Medicine and Pharmacy, 042122 Bucharest, Romania; 2 Department of Anatomy, Carol Davila University of Medicine and Pharmacy, 020021 Bucharest, Romania; mugurel.rusu@umfcd.ro

**Keywords:** traumatic brain injury, neuroinflammation, post-traumatic interval, biomarkers, microglia activation, cytokines

## Abstract

In forensic pathology, accurately estimating the time since injury is essential. Current histological and imaging approaches commonly miss subtle temporal changes, especially in deaths occurring within hours of injury. This review discusses the timing of neuroinflammation after traumatic brain injury and emphasizes possible markers for estimating the time of injury in forensic cases. Promising markers include microglial activation (allograft inflammatory factor 1 and transmembrane protein 119, detectable within 10 min to 2 h), β-amyloid precursor protein accumulation (20–35 min), high-mobility group box 1 translocation (2–6 h), cytokine fluctuations (IL-1β and TNF-α peak between 4 and 24 h, IL-6 shows delayed, extended elevation), sequential leukocyte infiltration (neutrophils from 2 to 48 h, lymphocytes after 3–5 days), blood–brain barrier breakdown markers such as fibrinogen and IgG leakage, loss of tight junction proteins (2–3 h), matrix metalloproteinase-9 activity (peaking at 24–48 h), and reactive astrocytosis with increased glial fibrillary acidic protein levels (from 12 to 24 h onward). The association between injury severity and inflammation is influenced by factors such as age, genetics (e.g., APOE ε4), coexisting conditions, and preexisting inflammation, which reduce the reliability of individual markers. A multiparametric approach may offer the best prospects to improve the accuracy of post-traumatic and post-mortem interval assessment in medicolegal cases.

## 1. Introduction

Traumatic brain injuries (TBIs) remain a major cause of death and long-term disability worldwide, with nearly 40 million cases annually [1], with many resulting in sudden or delayed death [1]. While historically caused mostly by high-speed motor vehicle crashes affecting young men, the epidemiology is changing, especially in high-income countries, with a notable rise in older adults. In addition, firearm-related suicides and homicides remain an important factor in certain regions, posing unique forensic challenges [2,3].

In forensic and legal medicine, accurately determining the “age” of a brain injury, especially the post-traumatic interval (PTI), is fundamental for reconstructing crime scenes and establishing legal responsibility. The forensic pathologist’s assessment of wound age can directly influence criminal charges (such as manslaughter versus murder) or civil cases (like wrongful death claims related to medical negligence). Therefore, any marker that objectively enhances PTI precision is extremely important, as it decreases dependence on subjective judgment. In forensic practice, the post-traumatic interval (PTI) refers to the time elapsed between injury and death. It must be distinguished from the post-mortem interval (PMI), the time elapsed between death and examination, as both influence molecular marker stability differently [4].

Classical methods, including histological techniques such as hematoxylin and eosin staining or imaging tests, often fail to detect subtle cellular changes in deaths that occur minutes to hours after injury. They also have difficulties in accurately determining the cause when the victim has experienced more than one traumatic event. Furthermore, distinguishing primary mechanical damage from secondary hypoxic–ischemic injury remains a major challenge, as both processes trigger overlapping necrotic, inflammatory, and apoptotic pathways [5,6]. Despite significant advancements in recent years, current forensic dating techniques remain imprecise. Conventional methods, such as neutrophil migration, have a wide temporal window, making them inadequate for precise dating. Although many immunohistochemical markers have been suggested, their practical usefulness is limited because supporting data are often based on small case numbers, varied methodologies, animal studies, or highly controlled environments [7,8]. Additionally, individual differences such as age, gender, underlying diseases, and post-mortem interval (PMI) may alter protein expression, making some markers unreliable without strict reference standards [5,9]. Critically, these confounding factors, including age, genetic background (e.g., APOE ε4 status), preexisting inflammatory conditions, and comorbidities, must be systematically considered when evaluating each individual marker, as they can significantly alter the kinetics and magnitude of neuroinflammatory responses, thereby affecting PTI estimation accuracy [10,11].

Neuroinflammation is one of the main drivers of secondary TBI. After the initial mechanical injury, a highly active and complex inflammatory cascade is triggered, characterized by activation of resident microglial cells, disruption of the blood–brain barrier (BBB), and infiltration of peripheral immune cells. This response is mediated by a complex network of cytokines, chemokines, and damage-associated molecular patterns (DAMPs) such as HMGB1 [12]. Although the goal of this reaction is to clear debris and promote repair, dysregulated neuroinflammation can cause additional cerebral edema, increased intracranial pressure, and neuronal apoptosis and necrosis, thereby worsening the severity of the initial injury and ultimately deteriorating the patient’s prognosis.

The main aim of this study is to provide a comprehensive review of the temporal dynamics of neuroinflammation following TBI and to evaluate potential markers that could improve the accuracy of post-traumatic interval estimation in fatal cases for forensic purposes. This review uniquely synthesizes current knowledge on the temporal dynamics of neuroinflammatory markers specifically for forensic post-traumatic interval estimation, providing a structured framework for marker selection and interpretation that has not been previously available in the forensic pathology literature. We acknowledge that this review synthesizes existing evidence rather than presenting novel empirical data, and that the proposed multiparametric approach requires prospective validation before clinical implementation. The transition from basic science markers to practical forensic application remains challenging, and we discuss these limitations throughout the manuscript.

## 2. Pathophysiology of Neuroinflammation in TBI

The pathophysiology of fatal TBI is a complex, evolving process that occurs in two main phases: an immediate, irreversible primary (traumatic) injury, followed by a delayed, potentially modifiable secondary injury cascade. The primary injury may be caused by contact phenomena such as skull fractures, contusions, and lacerations, as well as by indirect injuries like acceleration–deceleration [13]. The severity and distribution of these mechanical forces determine the extent and pattern of initial tissue damage. Various biomechanical studies have demonstrated that strain rate, magnitude, and duration each independently contribute to cellular injury thresholds. High-velocity impacts can generate pressure gradients that travel through brain tissue as stress waves, creating regions of positive and negative pressure that may surpass cellular and biomolecular tolerance limits [14]. The biomechanical deformation of neural tissue causes immediate cytoskeletal disruption, including damage to microtubules and neurofilaments, which occurs within milliseconds of impact [15]. These structural changes disrupt axonal transport and trigger a cascade of cellular and molecular disturbances that extend far beyond the initial mechanical injury [16].

Trauma-related injuries are generally classified as focal or diffuse, though they actually exist on a spectrum rather than as distinct categories [17]. Focal injuries include contusions, hematomas, and lacerations that affect specific brain regions, usually occurring at coup or contrecoup sites where the brain strikes or is struck by the skull. Contusions are commonly found at the frontal and temporal poles because of the irregular contours of the anterior and middle cranial fossae, respectively [18]. Diffuse axonal injury (DAI) is the main feature of diffuse TBI, characterized by widespread damage to axons across the cerebral hemispheres, corpus callosum, brainstem, and cerebellum [19]. It results from rotational acceleration or deceleration forces that produce shear, tensile, and compressive strains within white matter tracts, often at points where tissue properties change suddenly, such as gray-white matter junctions [20]. These mechanical forces cause axonal membrane permeabilization and damage to the cytoskeleton, initiating a process called “traumatic axonal injury,” which involves both immediate mechanical failure and delayed progressive degeneration [16]. Primary axotomy, the complete severing of axons, occurs only in the most severe injuries, whereas most external damage involves mechanoporation of the axolemma and subsequent ionic imbalance [21]. Axonal injury is closely associated with vascular damage; the mechanical forces frequently damage blood vessels, leading to immediate hemorrhage. Vascular damage ranges from small, microscopic petechial hemorrhages seen in mild TBI to large hematomas that require surgery in more severe cases. The cranial blood vessels show regional vulnerability, with bridging veins being especially prone to tearing during acceleration or deceleration injuries [22].

In regions subjected to forces exceeding cellular structural limits, primary mechanical trauma causes immediate necrotic cell death [23]. Necrosis primarily results from catastrophic membrane failure, leading to disruption of ionic homeostasis, cellular edema, and rapid ATP depletion [24]. The distribution of necrotic cell death closely matches the pattern of biomechanical injury, with the most significant tissue damage located at impact sites and along trajectories of maximum strain. Neurons are especially vulnerable to mechanical trauma compared to glial cells, with neuronal death occurring at lower strain thresholds than injuries to astrocytes or oligodendrocytes [25]. Necrotic cells release various intracellular molecules into the extracellular space, including ATP, high-mobility group box 1 (HMGB1), and other DAMPs, which trigger an immediate innate response and initiate the secondary injury cascade.

The secondary injury phase starts within minutes of trauma and can last for days, weeks, or even months. Excitotoxicity is one of the earliest and most severe mechanisms involved. Mechanical damage to neuronal membranes and energy failure lead to the uncontrolled release of excitatory neurotransmitters, such as glutamate, into the extracellular space [26]. Extracellular glutamate overstimulates ionotropic receptors such as N-methyl-D-aspartate (NMDA), α-amino-3-hydroxy-5-methyl-4-isoxazolepropionic acid (AMPA), and kainate receptors, leading to a large influx of calcium into neurons [27]. The highest glutamate release occurs at contusion margins or areas of secondary ischemia [28]. Intracellular calcium dysregulation contributes to secondary neuronal injury, as mechanical trauma causes immediate calcium influx through damaged membranes and delayed calcium entry via activated receptors and voltage-gated channels [29]. Overactivation of glutamate receptors and impaired calcium buffering result in sustained increases in cytoplasmic calcium, which activate many destructive enzymes like calpains, phospholipases, endonucleases, and nitric oxide synthase [30]. Additionally, mitochondrial calcium overload disrupts oxidative phosphorylation and triggers the mitochondrial permeability transition, initiating both necrotic and apoptotic cell death pathways [31,32].

Mitochondrial impairment is also a crucial aspect of secondary TBI, as mechanical trauma directly damages mitochondrial membranes and causes mitochondrial calcium overload [33]. As Xiong et al. have shown, mitochondrial respiration decreases immediately after TBI, with lasting deficits in oxidative phosphorylation that persist for weeks after the injury [34]. The dysfunction impacts multiple respiratory chain complexes, with Complex I showing particular vulnerability to trauma-induced damage. See Table 1.

The impairment of axonal mitochondria is especially important because they supply the high energy needed for the propagation of the action potential and synaptic transmission. Traumatic axonal injury causes disruptions in mitochondrial transport along axons, leading to the buildup of dysfunctional mitochondria at injury sites and energy failure in distant axonal segments [21]. These processes cause a delayed progression of axonal degeneration, which is typical of DAIs [33].

TBI increases oxidative stress through various mechanisms, including the production of reactive oxygen species (ROS) and reactive nitrogen species (RNS) from damaged mitochondria, the activation of oxidant-generating enzymes, and the depletion of natural antioxidant systems [59,60]. A detailed overview of the main factors driving oxidative stress after TBI is shown in Table 1. Superoxide radical, hydrogen peroxide, hydroxyl radical, and peroxynitrite build up in injured brain tissue, resulting in lipid peroxidation, protein oxidation, and DNA damage.

Lipid peroxidation, in particular, is a highly damaging outcome of oxidative stress because polyunsaturated fatty acids in neuronal membranes are very susceptible to free radical attacks [59]. The rapid spread of lipid peroxidation reactions through the neuronal membrane damages its structure, disrupts ion channels and receptors, and produces toxic aldehydes such as 4-hydroxy-2-nonenal and malondialdehyde. These products further impair cellular function and serve as biomarkers of oxidative injury. Protein carbonylation and nitration can alter key cellular protein functions, including enzymes involved in energy metabolism, cytoskeletal components, or ion channels [61]. Proteomic studies of injured brain tissue have identified hundreds of proteins affected by oxidation, with significant consequences such as impaired glucose metabolism (proteins like pyruvate dehydrogenase, ATP synthase, cytochrome c oxidase Va, enolase 1) [62,63], disrupted protein degradation pathways (ubiquitin carboxy-terminal hydrolase L-1, creatine kinase BB, heat shock cognate 71) [64,65], or disturbed cellular signaling (Synapsin I, SAP-97, PSD-95, α-enolase) [45,50,62]. DNA oxidation can cause mutagenic lesions that, if unrepaired, can lead to cell death; 8-hydroxy-2′-deoxyguanosine is a common marker.

The BBB is a specialized part of the brain’s microvasculature, consisting of several closely connected cell types. The most important (and well known) are brain endothelial cells, which form the inner lining of CNS capillaries. They feature continuous, highly specialized junctional complexes (such as adherens and gap junctions), have low levels of vesicular transport, and contain a rich array of transporters and enzymes that carefully regulate molecular movement between the blood and brain tissue [66,67]. This endothelial layer is surrounded by two other types of perivascular cells—pericytes and astrocytes. Pericytes are embedded in the shared basement membrane and are directly adjacent to endothelial cells. In vivo, loss-of-function studies have shown that a deficiency of pericytes increases BBB permeability to water, small tracers, and large tracers by increasing endothelial transcytosis, altering endothelial BBB-specific gene expression, and shifting the polarization of surrounding astrocyte endfeet. Astrocytes, by extending their endfeet, nearly fully encase CNS microvessels, creating a physical and biochemical interface. They release growth factors that promote and maintain tight junction protein expression in endothelial cells, helping preserve barrier selectivity and water/ion balance via aquaporin-4. TBI damages BBB integrity through multiple mechanisms, including direct mechanical injury to blood vessels, activation of MMPs, oxidative stress, and effects of inflammatory mediators [68].

The disruption generally occurs in three stages: an initial very early opening within 30 min, followed by a partial recovery, and a second, relatively early opening within a few hours after the trauma, leading to another partial recovery and a third opening several days later. Within minutes after trauma, some studies have observed a transient opening. Certain fluid-percussion and closed-head injury studies have shown that BBB breakdown to circulating proteins such as albumin and HRP can happen within 3–13 min after impact and may be widespread across both hemispheres, as a general response to trauma [69,70]. The greatest extravasation occurs within the first hour, followed by partial restoration within 30–60 min in many areas, except at the impact site, which remains permeable for a longer period. In the second phase, which was studied in more detail, it was shown that, around four hours after injury, the BBB is permeable to both large (horseradish peroxidase, around 44 kDa) and small tracers (biotin–dextrin–amine, 3 kDa) [71,72]. Over the next 12–24 h, BBB permeability is partially restored (especially for large molecules), and it increases again (particularly for small solutes) at 2–5 days, being associated with neuroinflammation, endothelial transcytosis, tight junction alterations, and vasogenic edema [71,72].

Disruption of tight junction proteins plays a vital role in the breakdown of the BBB. Many experimental studies show decreased expression and abnormal cellular localization of occludin, claudin-5, or zonula occludens after TBI. For example, Web et al. have demonstrated that in a rat TBI model, mRNA and protein levels of claudin-5, occludin, and zonula occludens-1 in the peri-contusional cortex significantly decrease after injury. These changes are linked to increased BBB permeability, as evidenced by Evans blue, directly connecting tight junction loss to BBB opening [73]. Similarly, Kawoos et al. found, in an experimental blast TBI study in rats, variable reductions in the same proteins at 2–3 h and 1 day post injury, coinciding with increased leakage of a 40 kDa tracer [74]. A more recent study identified claudin-1 induction in brain endothelium as a pathogenic mechanism: increased claudin-1 levels were associated with downregulation of claudin-5, occludin, and zonula occludens-1, leading to decreased transendothelial electrical resistance and increased permeability. Knockdown of claudin-1 preserved junctional proteins and maintained barrier integrity [75]. BBB dysfunction allows the infiltration of the brain by peripheral immune cells, plasma proteins, and potentially neurotoxins [68]. Albumin extravasation activates astrocytes and triggers inflammatory responses via TGF-β signaling pathways [76]. Additionally, infiltration of peripheral macrophages and lymphocytes contributes to neuroinflammation and may influence both injury progression and recovery [77].

### 2.1. The Neuroinflammatory Continuum

While this section details the mechanotransduction mechanisms linking primary mechanical injury to secondary inflammatory responses, readers primarily interested in forensic marker applications may proceed directly to Section 3. The mechanotransduction pathways described here provide important context for understanding why certain markers appear at specific time points post injury. Still, current technology does not permit post-mortem assessment of mechanotransduction states. The forensic utility of this section lies in understanding the biological rationale for marker temporal profiles rather than direct measurement of these pathways.

The conceptual division between primary and secondary brain injury, while useful for organization, oversimplifies the complex reality of TBI pathophysiology, which actually involves a continuous spectrum of overlapping processes initiated at the moment of impact [78]. The primary mechanical insult triggers an immediate and intense cellular and biomolecular response, blurring the line between direct mechanical damage and secondary effects, with mechanical strain activating signaling pathways, altering gene expression, and initiating inflammatory cascades within minutes [24]. The main link between primary and secondary injury mechanisms is mechanotransduction, the process by which cells convert mechanical stimuli into biochemical signals. Stretch-activated ion channels, integrin-mediated mechanosensing, and deformation of the primary cilium all contribute to cellular responses to mechanical trauma, with activation of mitogen-activated protein kinase (MAPK) pathways, nuclear factor-κB (NF-κB), and hypoxia-inducible factors occurring rapidly after injury [29]. See Table S1 for the main mechanisms involved in mechanotransduction after TBI.

One of the key components in mechanotransduction-associated neuroinflammation is the Piezo channels. These are a family of large mechanosensitive ion channels embedded in the cell membrane, which open when the membrane is mechanically deformed (stretched, bent, or under pressure). When they open, positively charged ions flow into the cell, transforming mechanical force into electrochemical signals. In microglia, Piezo1 channels are involved in migration and pro-inflammatory cytokine production, acting as gatekeepers of the microglial immune response [79]. In astrocytes, Piezo1 channels play bidirectional roles—inflammation increases their sensitivity, and Piezo1 activity influences astrocyte behavior, reinforcing neuroinflammatory circuits [80].

While these mechanosensitive pathways cannot be directly assessed post mortem, their downstream effects, such as cytokine production patterns and inflammatory cell activation, represent the measurable forensic markers discussed in Section 3. Understanding mechanotransduction helps explain why certain markers appear within minutes (e.g., microglial morphological changes) while others require hours to days (e.g., leukocyte infiltration, astrocytic GFAP upregulation) [81,82,83].

Neuroinflammation after TBI shows complex spatio-temporal dynamics, with both beneficial and harmful effects that evolve over time. It involves many cell types and can both worsen damage and promote repair, sometimes occurring simultaneously in different brain regions [35,82]. In the acute phase, innate immune activation increases edema, oxidative stress, excitotoxicity, and cell death, while also aiding debris clearance, infection containment, angiogenesis, and early tissue repair [84,85,86]. As the response shifts into the chronic phase, ongoing activation of microglia and astrocytes, sustained cytokine and chemokine production, and continued leukocyte infiltration contribute to progressive neurodegeneration, seizures, cognitive decline, and neuropsychiatric syndromes [85,87,88]. The same mediators, such as IL-6, CCL2, and TNFα, can be neuroprotective when transient or low-level, or neurotoxic when excessive or prolonged [84,89].

Microglia act as rapid sentinels after TBI, quickly shifting from a homeostatic to a reactive state within minutes to hours. During this process, they shape much of the downstream cytokine and chemokine environment. In the uninjured brain, they have small bodies and highly branched processes that constantly monitor the parenchyma [90]. After TBI, they quickly undergo morphological changes toward a hypertrophic or amoeboid shape, indicating increased phagocytic activity [90,91]. Studies on human cadavers have shown localized activation and erythrocyte phagocytosis in the contusion zone even with survival times of only a few minutes, suggesting an almost immediate response to injury [92]. Using a rat model, Caplan et al. observed amoeboid microglia in the hippocampus and thalamus within 24 h after TBI, with increased density and an activated phenotype that persisted for weeks [91]. These structural changes are linked to enhanced motility and process reorientation toward injury sites, enabling microglia to contact damaged neurons, myelin, or blood-derived elements [9,90,93]. Their activation leads to increased phagocytosis of cellular debris and synaptic components. Around a week later, microglia show enhanced phagocytic activity and begin to engulf synapses, including the formation of satellite microglia tightly attached to neuronal cell bodies and dendrites. This can contribute to synaptic loss and circuit dysfunction. While this process is essential for clearing debris, excessive or inappropriate engulfment of healthy synapses and neurons has been increasingly associated with cognitive deficits or maladaptive plasticity following TBI.

**Figure 1:**
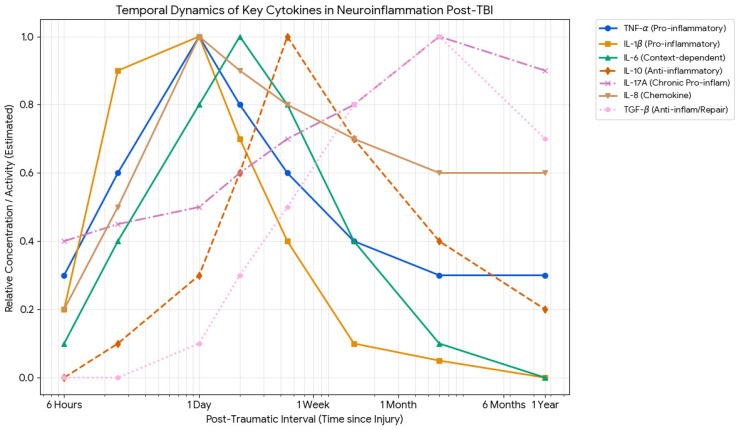
Temporal dynamics of key cytokines in neuroinflammation after TBI. Composite representation based on experimental and clinical studies [98,99,100,101,102,103,104,105,106,107,108,109,110], including rodent controlled cortical impact models, human CSF/serum studies, and post-mortem tissue analyses. Individual studies used varying injury models, species, and measurement methods. Axes: X-axis represents time post injury (hours to days); Y-axis represents relative expression level (normalized, arbitrary units). Actual absolute values vary substantially across studies depending on methodology, injury severity, and biological sample (tissue, CSF, serum). Limitations: This figure represents a synthesis of available data and should be interpreted as illustrating general temporal patterns rather than precise quantitative values. Individual patient/case variability is substantial.

When triggered by injury-related cues or by early cytokines such as TNFα or IL-1β, microglia release a wide array of secondary cytokines and chemokines in a concentration- and time-dependent manner, far exceeding what neurons or astrocytes release [94]. Transcriptomic profiling of microglia isolated 2–60 days after injury reveals dynamic waves of inflammatory gene expression, with an initial decrease in homeostatic genes and a later development of a specialized inflammatory state that combines both pro- and anti-inflammatory signatures [95] (see Figure 1). These states include upregulation of interferon-related, NF-κB-driven, and neurodegenerative-associated programs, which shape the overall neuroinflammatory cascade and influence neuronal survival, synaptic plasticity, and neurogenesis [88,96,97].

Pro-inflammatory cytokines play multiple roles in injured brain tissue, including exacerbating excitotoxicity by altering glutamate receptor expression, increasing BBB permeability, recruiting peripheral immune cells, and promoting cell death pathways [111]. The inflammatory response begins with an initial innate immune phase, followed by a delayed adaptive immune response involving T and B lymphocytes, which can persist for months after injury [77] and contribute to ongoing neurodegeneration [16]. Post-mortem studies of TBI survivors show microglia activation years after injury, with chronic inflammatory changes observed in both white and grey matter [16]. Positron emission tomography imaging with ligands targeting the translocator protein (TSPO), a marker of microglial activation, has demonstrated chronic neuroinflammation even 17 years post TBI [112].

The mechanisms maintaining chronic neuroinflammation after TBI involve multiple positive feedback loops and the failure of normal resolution pathways [113]. Cellular damage releases DAMPs, such as extracellular DNA/RNA, HMGB1, and heat shock proteins, which bind pattern-recognition receptors, such as Toll-like receptors, on microglia, astrocytes, or endothelial cells. This activates NF-κB/MAPK signaling and drives the production of IL-1β, IL-6, TNF-α, chemokines, ROS, and NO, which further damage neurons and the BBB, creating more DAMPs and sustaining microglial activation. Immediately after TBI, microglia may adopt reparative, M2-like states. Still, over time, there is a shift toward persistent M1-like, pro-inflammatory microglia with decreased phagocytic activity and increased production of TOS, NO, and cytokines [81]. This ongoing bias toward the M1 state prevents the normal resolution of inflammation and promotes continuous neurodegeneration and demyelination. TBI also impairs autophagic flux. Altered autophagy reduces DAMP clearance and prevents degradation of innate components, such as the NLRP3 inflammasome, leading to exaggerated type-1 interferon production and sustained inflammasome signaling, causing sustained cytokine release [114]. Pro-inflammatory microglia also exhibit mitochondrial dysfunction and shift toward glycolysis, which supports increased ROS and cytokine production but hampers homeostatic functions such as surveillance, phagocytosis, and resolution. Overall, these mechanisms promote chronic, non-resolving neuroinflammation.

Senescent cells, which build up in injured brain tissue, represent another mechanism linking acute injury to chronic inflammation and neurodegeneration [115]. Cellular senescence, marked by permanent cell cycle arrest and the release of inflammatory mediators (the senescence-associated secretory phenotype, SASP), increases after TBI and contributes to the ongoing inflammatory state [116].

### 2.2. The Interaction Between Injury Severity and Inflammatory Response

Both experimental and clinical studies have shown that greater tissue damage from TBI is associated with a larger, longer-lasting inflammatory response. For example, in a rodent study by Lagraoui et al., severe TBI led to an amplified response that persisted for at least 21 days. In contrast, in milder TBI, neuroinflammation nearly returned to baseline within 10 days [117]. Michalovicz et al., also in a rodent study, used a projectile-concussive model to induce mild versus moderate-severe TBI, finding that the latter results in greater neurodegeneration and widespread neuroinflammation across the cortex, hippocampus, thalamus, and cerebellum. In contrast, milder TBI causes more localized and less intense neuroinflammation [118]. Clinically, higher systemic cytokine levels, such as IL-6 or IL-10, have been associated with higher overall Injury Severity Score and increased mortality risk, supporting a link between injury severity and the inflammatory response [119]. Another study by Johnson et al. demonstrated that serum proteins and cytokines, such as ASC, IL-18, TNFα, IL-4, and IL-6, increase after TBI and correlate with GCS scores and overall outcomes [120]. However, this relationship is not always straightforward. For instance, in a large polytrauma cohort, patients with isolated severe TBI had lower IL-6 and IL-10 levels than polytrauma patients without TBI, despite having similar or higher ISS [119]. The same study observed that as head AIS severity increased, overall cytokine expression tended to decrease, and the IL-6/IL-10 ratio suggested a somewhat suppressed pro-inflammatory response, indicating a potential immunomodulatory or immunosuppressive effect of severe TBI on systemic inflammation [119]. Samanta et al. studied immune endotypes in patients with moderate-to-severe TBI and identified “early inflammatory” and “pauci-inflammatory” phenotypes that were not explained by baseline injury severity scores. Patients in the early-inflammatory group had higher levels of IL-6, IL-15, and MCP-1 and worse outcomes, but did not systematically exhibit greater anatomical severity than pauci-inflammatory patients [121].

Numerous studies indicate that the injury–inflammation link is influenced by factors such as age, genetics, prior immune challenges, extracranial injuries, infections, metabolic syndrome, and other comorbidities. These elements can modify the immune response during TBI, influencing the severity and nature of post-traumatic inflammation. Refer to S2 for additional information.

## 3. Forensically Relevant Markers for PTI Estimation

The earliest detectable changes following TBI occur at the cellular and molecular level, often before conventional histological changes become visible. These hyperacute markers are especially useful in cases where death occurs shortly after injury or when determining whether an individual was alive at the time of the traumatic event.

### 3.1. Microglial Activation and Morphological Changes

Microglial responses are among the earliest cellular reactions to TBI and can be detected relatively quickly using immunohistochemistry. In human forensic material, Lier et al. examined eight fatal TBI cases with survival times ranging from minutes to 7 days and showed activated, Iba1+ microglia near contusions in a case with a survival time of only 10 min, whereas control samples showed only non-activated microglia [92]. The same study showed progressive changes in microglial morphology and antigen expression (including ferritin and GPX1 upregulation) with increasing time to trauma survival [92]. Another study, done by Bohnert et al., assessed TMEM119, a microglia-specific marker, in 25 lethal TBI cases, and found a significantly increased number of TMEM119+ microglia, even in acute deaths with survival times less than 2 h, compared to cardiovascular controls, with survival-time-dependent changes in TMEM119, CD206, CCR2-positive cells in the cortex, white matter, brainstem, and cerebellum [122]. Flow cytometry studies show that microglia activate within minutes after TBI, with increased cell numbers and altered surface marker profiles and morphologies evident by 24 h, especially in the ipsilateral hemisphere [123]. Although several groups have proposed semi-quantitative scoring systems or morphometric pipelines to classify microglial states (ramified, hypertrophic, amoeboid, rod-like) after TBI, reported temporal thresholds (e.g., exact hour ranges for each morphology) have substantial variability across models, species, brain regions, and injury severity, and therefore are not standardized enough to support precise PTI estimation for routine forensic practice [90,93,124,125]. Advanced age is associated with baseline microglial priming and altered morphology, potentially confounding interpretation. Preexisting neurodegenerative conditions (Alzheimer’s, Parkinson’s) show chronic microglial activation. Systemic infections can induce microglial activation independent of TBI. Post-mortem interval affects immunohistochemical detection sensitivity [98,126].

An important confounding factor, which shoul be explicitly addressed when interpreting microglial markers in the context of TBI is the contribution of hemorrhage-associated infiltrating myeloid cells. Almost always, TBIs cause some degree of vascular damage, ranging from microscopic petechial hemorrhages to large contusional hematomas, which in turn leads to immediate disruption of the blood-brain barrier and rapid invasion of peripheral blood-derived monocytes and macrophages into the brain parenchyma [99,100]. These infiltrating monocyte-derived macrophages share many surface markers with resident microglia, including Iba1, CD68, and CX3CR1, making the two populations largely indistinguishable when using conventional immunohistochemistry [99,101]. Markers previously considered to be specific to microglia, such as TMEM119 and P2RY12,were shown to lose their discriminatory efficacy in the injured brain. For example, TMEM119 protein expression is downregulated in reactive microglia to levels comparable to those of blood-borne macrophages, thereby failing to differentiate the two myeloid populations after TBI [102]. Fate-mapping studies have revealed that infiltrating monocytes can acquire de novo expression of canonical microglial markers, such as TMEM119, P2RY12, and Sall1, and eventually become phenotypically indistinguishable from embryonic microglia using conventional approaches [103,104]. This phenotypic convergence means that studies quantifying “microglial” activation after hemorrhagic TBI using markers such as Iba1 or CD68 inevitably capture a mixed population of resident microglia and infiltrating macrophages, a limitation that affects the interpretation of virtually all immunohistochemical studies discussed in this review.

The functional implications of this microglia-macrophage overlap are directly relevant to forensic PTI estimation. Infiltrating MDMs may actively influence the neuroinflammatory milieu through crosstalk with resident microglia, promoting type I interferon responses and altering microglial activation profiles, as demonstrated by single-cell RNA sequencing studies in CCR2-deficient TBI models [105]. Moreover, MDMs persist in the brain parenchyma for at least 8 months after TBI, retaining phagocytic activity and displaying a distinct transcriptomic signature associated with aging and disease, which may confound the interpretation of chronic neuroinflammatory changes [106]. In the specific context of intracerebral hemorrhage, both microglia and infiltrating macrophages are recruited to the perihematomal region, where they participate in hematoma clearance through erythrophagocytosis, produce pro-inflammatory cytokines (IL-1β, TNF-α, IL-6), matrix metalloproteinases, and reactive oxygen species, and undergo phenotypic polarization that overlaps temporally with the marker dynamics described above [106]. Flow cytometry using CD45 expression levels (CD45lo for microglia versus CD45hi for peripheral macrophages) or advanced techniques such as single-cell transcriptomics and genetic fate-mapping remain the most reliable approaches for discriminating these populations [101], but these methods are largely impractical in routine forensic post-mortem examination. Therefore, until reliable immunohistochemical panels capable of distinguishing microglia from infiltrating macrophages in autopsy tissue become available, the temporal profiles of “microglial” markers reported herein should be interpreted with the caveat that they likely reflect the combined response of both resident and blood-derived myeloid populations, particularly in hemorrhagic TBI cases where vascular disruption facilitates early and significant macrophage infiltration.

Much of the reported temporal kinetics for microglial activation and cytokine responses derives from experimental (mainly rodent) models; rodent-to-human extrapolation requires temporal scaling, and the indicated time windows should be interpreted as approximate forensic ranges rather than exact chronological thresholds.

### 3.2. Mechanotransduction and Early Axonal Damage

The immediate mechanical response to TBI, involving inertial acceleration–deceleration forces, produces shear, tensile, and compressive strains within the brain, deforming tissue and placing long-tract axons at risk of shear and stretch injury [107]. The distortion of the axonal cytoskeleton disrupts normal axonal transport. It leads to the accumulation of transported proteins in the injured segments, causing axonal swelling and altered neuronal homeostasis [108], with βAPP being the most studied. Human autopsy studies have shown βAPP accumulation within 2–3 h (sometimes even earlier) after TBI. For example, Hortobágyi et al. found that in adults with severe head injury, βAPP+ axons were detected using antigen retrieval techniques in cases surviving for 35–60 min or less.

In contrast, cases with survival of less than 30 min were negative [109]. Nedić et al. observed βAPP+ axons in adult and pediatric cases with a survival time of 20–35 min after acceleration–deceleration head injuries [110]. Al-Sarraj et al., in a larger cohort of subjects, demonstrated βAPP+ axons in 35 of 37 cases of road traffic deaths with survival times of less than 30 min [108]. However, βAPP is a general marker of impaired axonal transport, not specific to traumatic axonal injuries, as it has also been identified in axonal injuries caused by hypoxic–ischemic or other metabolic insults. Studies comparing traumatic with vascular/ischemic axonal injuries have shown, however, that βAPP staining patterns and distributions differ between mechanically sheared axons and ischemic damage, though there is substantial overlap.

Additionally, pattern-based approaches (e.g., axons aligned with fiber tracts versus scattered) may help differentiate between traumatic and hypoxic axonal injuries in some cases, but overall specificity remains low [127,128]. Other axonally transported proteins, such as neurofilament (NF-68), SNAP-25, chromogranin A, or cathepsin D, may also accumulate at sites of axonal injury and can be detected immunohistochemically. Still, they generally have lower sensitivity and specificity than βAPP [129]. From a practical forensic standpoint, βAPP should be interpreted in a pattern-based manner: tract-aligned axonal swellings/varicosities and retraction bulbs favor mechanically induced diffuse axonal injury, whereas more diffuse or watershed/perivascular staining patterns are more compatible with hypoxic–ischemic injury; therefore, βAPP is best interpreted in combination with mechanotransduction readouts and BBB/vascular markers (e.g., IgG/fibrinogen extravasation, tight junction proteins, MMP-9).

From a practical forensic standpoint, βAPP should be interpreted in a pattern-based manner: tract-aligned axonal swellings/varicosities and retraction bulbs favor mechanically induced diffuse axonal injury, whereas more diffuse or watershed/perivascular staining patterns are more compatible with hypoxic–ischemic injury. Therefore, βAPP is best interpreted in combination with mechanotransduction readouts and BBB/vascular markers (e.g., IgG/fibrinogen extravasation, tight junction proteins, MMP-9) [110,130,131].

### 3.3. Damage-Associated Molecular Patterns

HMGB1 is a nuclear protein that becomes a DAMP when it translocates into the cytoplasm and is released extracellularly, activating TLR4/RAGE and cytokine cascades [132,133,134]. In brain injury models, nuclear-to-cytoplasmic translocation and release occur within minutes to a few hours. Rodent models of TBI have shown nuclear loss and cytoplasmic accumulation beginning at 6 h and persisting for days. The cytosolic fractions are elevated from 2 to 24 h and later [135,136,137,138]. In brains with hypoxic–ischemic injuries, neuronal HMGB1 translocation was seen from 0 to 2 h, and extracellular detection from 2 to 6 h [139,140]. In subarachnoid hemorrhage, neuronal translocation was also observed as early as 2 h after the onset of hemorrhage, with increased mRNA/protein levels and downstream NF-κB/IL-1β activation [141]. Their potential usefulness is relatively limited to PTI, as its pattern varies with injury type, severity, age, and cell death mode (necrosis vs. apoptosis), leading to inconsistent early profiles across studies [132,133,136]. HMGB1 is also affected by systemic trauma, sepsis, and peripheral organ injury, complicating its interpretation [137,142,143]. HMGB1 is a relatively non-specific DAMP: in early post-injury deaths, it may also be increased or translocated in non-traumatic hypoxic–ischemic injury and systemic inflammation, and HMGB1 alone cannot reliably discriminate traumatic from non-traumatic mechanisms. Systemic trauma and sepsis elevate peripheral HMGB1, potentially confounding CNS interpretation. Age affects HMGB1 dynamics. The mode of cell death (necrosis releases HMGB1 rapidly; apoptosis retains it) creates variable profiles. Post-mortem autolysis affects the nuclear–cytoplasmic distribution [134,144,145].

### 3.4. Temporal Profiles of Key Cytokines

Experimental and clinical TBI data have shown rapid IL-1β upregulation within hours, peaking on the first day and contributing to a narrow treatment window [146,147,148]. In the cortex and hippocampus, IL-1β peaks between 4 and 24 h, with elevations persisting for days. Blood and CSF studies in human TBI show increased IL1β and IL-1ra in the acute (<24 h) and subacute (<7 days) phases, often associated with BBB disruption and worse outcomes [149,150,151]. Similarly, TNFα appears very early after TBI, with cortical mRNA and protein detectable from 1 to 4 h, sometimes peaking before 12 h and declining thereafter, though in other models it remains elevated for a few days [152,153,154,155]. In contrast, human serum TNFα levels rise acutely after TBI and remain chronically elevated, correlating with severity and overall outcomes [120,156]. IL-6 typically shows a slightly delayed (24 h) but more prolonged rise and is considered the most consistently increased acute marker, with elevated blood levels for months [151]. IL-1-dependent chemokines (CCL2, CCL3, CCL4) show strong temporal regulation: in mouse closed-head injury, Ccl3/Ccl4 peak at three h and Ccl2 at nine h, then returning toward baseline by 24–72 h [146]. For forensic purposes, the stereotyped early appearance (2–6 h) and changing cellular/spatial patterns over days mean these markers can support broad PTI binning (e.g., <6 h, 6–24 h, 1–3 days, >3 days). However, expression is influenced by injury severity, systemic inflammation, BBB status, and treatment options [146,149,150,151].

Preexisting systemic inflammation, infection, or sepsis significantly alter cytokine baselines. Medical interventions (such as corticosteroids and hypothermia) can suppress cytokine responses. Age affects cytokine kinetics (the elderly show delayed peaks). Genetic polymorphisms in cytokine genes create inter-individual variability. Post-mortem degradation preferentially affects cytokines compared to structural proteins. Much of the reported temporal kinetics of microglial activation and cytokine responses derives from experimental (mainly rodent) models; rodent-to-human extrapolation requires temporal scaling, and the indicated time windows should be interpreted as approximate forensic ranges rather than exact chronological thresholds [157,158,159].

### 3.5. Leukocyte Characteristics

The ordered arrival of neutrophils, monocytes/macrophages, and then lymphocytes is one of the best-known and most widely used methods for determining PTI. Neutrophils are the first infiltrating leukocytes, arriving within minutes [160] into the subarachnoid/subdural spaces and peaking approximately 2 h later in animal models. The parenchymal infiltration peaks at around 24–48 h and declines over 7 days [9,161]. MPO activity increased at 6 h, peaked at 24–48 h, and returned to baseline by 72–168 h, depending on the region [161,162,163]. CD45+ and CD11b+ infiltrating myeloid cells in controlled cortical impact peak at around 18–24 h and remain elevated for several days, returning to baseline in about 2 months [9,164]. Regarding lymphocytes, human biopsy/post-mortem samples show no CD3+, CD4+, or CD8+ T cells within 24 h after TBI, but CD3+ and CD4+ T cells are present in essentially all samples at 3–5 days, with CD8+ T cells present in only 75% of cases. CD3+ cells persist for more than 26 days [9]. Systemic infection accelerates leukocyte infiltration. Immunosuppression (medical or age-related) delays infiltration. Preexisting inflammation may show baseline parenchymal leukocytes. Corticosteroid treatment suppresses infiltration.

### 3.6. BBB Breakdown Markers

Both human and animal studies have shown the presence of serum proteins in the parenchyma within hours after TBI. Swine and rodent contusion models have demonstrated multifocal IgG and fibrinogen leakage from 6 h to approximately 72 h after trauma, even in the absence of overt hemorrhage [74,165]. Human autopsy studies reported perivascular and parenchymal fibrinogen/IgG in 40–50% of acute TBI deaths at 10–13 days, and in nearly half of the patients with survival times over 1 year, suggesting an early onset and also a remarkable chronic persistence of BBB leakage [166,167]. Fibrinogen deposits are larger in acute TBI than in long-term survivors and are correlated with microglial activation and decreased neuronal density [168].

Blast and focal ischemia models show rapid reduction of claudin-5, occludin, and ZO-1 within 2–3 h, paralleling increased permeability to 40 kDa tracers, with near-normalization by 3 days in blast TBI [74], and a biphasic leak (peaks at 3 h and 72 h) in ischemia–reperfusion models [73]. After contusional TBI, TJ downregulation tracks the extravasation of Evans blue/albumin over 24–96 h [73]. Recent studies have also shown maladaptive claudin-1 induction, which suppresses claudin-5/ZO-1 and sustains leakiness in chronic injury [75,169].

MMPs also exhibit distinct temporal patterns. Experimental TBI and stroke models have shown early MMP-9 upregulation (2–6 h), with peak activity around 24–48 h, coinciding with TJ loss, laminin degradation, and vasogenic edema [170,171,172,173]. Human contusional TBI microdialysis studies have detected increased MMP-9 levels before 24–74 h, with the highest concentrations in the pericontusional areas, followed by a substantial decline thereafter, while other MMPs show variable or modest changes [174]. Serum MMP-7 and, to a lesser extent, MMP-1 and MMP-2, are correlated with MRI-quantified BBB permeability, suggesting the potential usefulness of systemic MMPs as peripheral biomarkers for BBB dysfunction [175]. Hypertension and diabetes cause baseline BBB dysfunction. Hypoxic–ischemic injury also disrupts the BBB. Post-mortem autolysis affects tight junction protein immunoreactivity. Systemic inflammation can increase circulating MMPs [176].

### 3.7. Reactive Astrocytosis and GFAP

**Figure 2:**
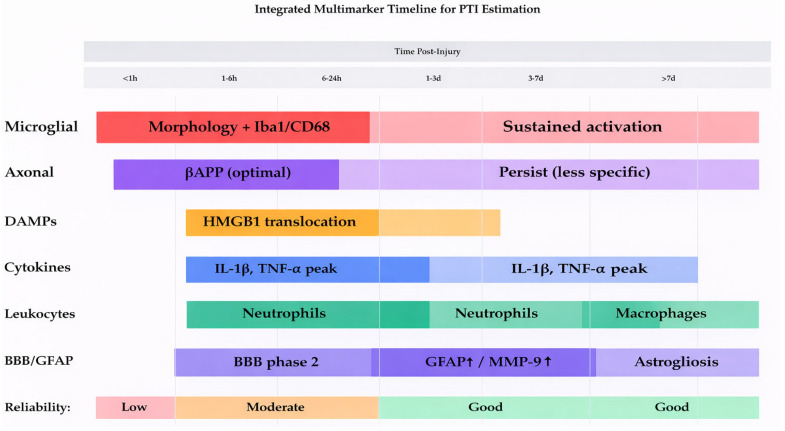
Integrated multimarker timeline for PTI estimation.

Reactive astrocytosis and GFAP (including GBDPs) serve as reliable markers for subacute to chronic PTI, especially if the estimated PTI is longer than 12–24 h. The 12–24 h threshold for GFAP elevation refers specifically to detectable tissue GFAP via immunohistochemistry in autopsy specimens. In contrast, serum GFAP can be detected earlier (within 1–6 h) due to blood–brain barrier disruption, releasing pre-formed protein. Forensic pathologists should note that autopsy-based assessment relies on tissue markers, making the 12–24 h tissue threshold most relevant for post-mortem evaluation (see Table 2 and Figure 2). However, responses differ depending on injury type (focal versus diffuse), severity, age, and sex, and some astrocytes become “atypically” reactive without significant GFAP upregulation [177,178,179]. In focal and stab-wound models, GFAP+ reactive astrocytes appear within 24 h, increase at 3–7 days, and remain elevated for more than 28 days, especially near the core of the injury [180,181,182]. Diffuse or mild TBI shows more modest GFAP increases, with subsets of astrocytes exhibiting only 2–3-fold GFAP elevations compared with higher levels in focal injuries [177,178]. TBI also triggers GFAP breakdown, with the appearance of GFAP breakdown products (GBDPs) via calpains and caspase-6, with fragments 38–46 kDa appearing from day 1, rising through 14–28 days, and marking delayed astrocyte injury [183]. Serum and CSF GFAP levels also rise within hours after TBI, reaching peak values over the first 24–48 h and remaining elevated for several days; there is a positive correlation between serum levels and TBI severity, CT lesions, neurosurgical intervention, and long-term outcome [119,183,184,185].

Preexisting astrogliosis from neurodegenerative disease, prior TBI, or stroke creates elevated baselines. Age affects astrocyte reactivity patterns. Hypoxic–ischemic injury also induces GFAP upregulation, limiting specificity for traumatic injury. Key distinguishing features, differentiating traumatic from hypoxic–ischemic injury include (1) β-APP accumulation pattern: traumatic axonal injury shows characteristic varicose axonal swellings along white matter tracts, while hypoxic–ischemic injury produces more diffuse neuronal β-APP. (2) Microglial activation distribution: TBI shows focal activation at injury sites versus global activation in hypoxia. (3) Temporal sequence: traumatic inflammation follows biomechanical injury distribution, while hypoxic patterns follow vascular territories [200,201,202,203]. Mixed injuries remain challenging and require combined marker assessment with careful correlation to gross pathological findings.

## 4. Proposed Multiparametric Decision Framework for PTI Estimation

**Figure 3:**
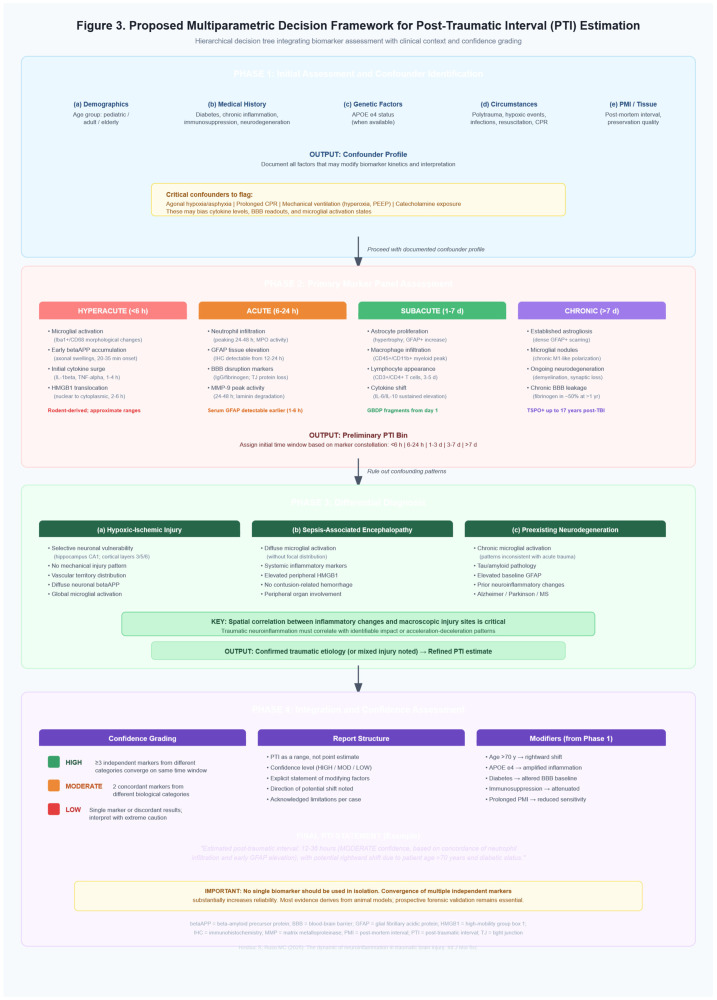
A multiparametric approach for PTI Estimation.

Given the limitations outlined above and the complexity of neuroinflammatory responses following TBI, we propose a structured multiparametric decision framework for forensic PTI estimation. This approach integrates multiple biomarker categories with contextual and clinical information to improve estimation accuracy while accounting for individual variability. The framework follows a hierarchical decision tree structure organized into four sequential evaluation phases: (1) Initial assessment and the identification of confounders; (2) Primar marker panel assessment; (3) Differential diagnosis and (4) Integration and confidence assessment. See Figure 3 for details.

## 5. Limitations

Biological variability, driven by factors such as advanced age, comorbidities (e.g., diabetes or chronic alcohol use), and genetic predispositions (e.g., APOE ε4), significantly influences the kinetics and magnitude of the neuroinflammatory response. This results in “delayed” or “attenuated” signatures that make post-traumatic interval (PTI) estimation difficult. These challenges are exacerbated by technical issues, including inconsistent antibody standards and the adverse effects of extended post-mortem intervals and tissue autolysis, which tend to reduce sensitive markers such as cytokines while preserving structural proteins. Furthermore, interpreting neuroinflammation is complicated by the difficulty in differentiating trauma from other patterns caused by hypoxic–ischemic events, systemic infections, or preexisting neurodegenerative conditions. The existing literature often suffers from small sample sizes, selection bias, subjective semi-quantitative scoring with inter-observer variability, and a general lack of thorough external validation, emphasizing the importance of a cautious, multi-marker approach in medicolegal contexts.

Important forensic confounders, such as agonal hypoxia/asphyxia, prolonged resuscitation (CPR), mechanical ventilation (including hyperoxia and PEEP-related effects), and catecholamine surges/vasopressor exposure, can modulate cytokine concentrations, BBB permeability readouts, and microglial activation states. These factors may therefore bias PTI estimation if not documented and accounted for, reinforcing the need for multiparametric interpretation and case-specific context.

Much of the existing literature is derived from animal models or clinical TBI cohorts with known injury timing, which may not directly translate to forensic contexts where injury timing is unknown. Prospective validation studies using forensic autopsy cases with documented injury times are essential.

Age-related baseline neuroinflammation, genetic polymorphisms (particularly APOE ε4), preexisting conditions (diabetes, hypertension, neurodegenerative diseases), and chronic medication use can alter marker expression and timing. Preexisting neuroinflammation from conditions such as Alzheimer’s disease, multiple sclerosis, or chronic infections may obscure or accelerate trauma-induced changes.

Cytokines (particularly IL-1β and TNF-α) show significant degradation within 24–48 h post mortem; GFAP and β-APP demonstrate relative stability for 24–72 h under standard autopsy conditions; microglial morphology is preserved for 24–48 h but autolysis limits later assessment. Refrigeration extends marker stability.

Antibody clone selection, tissue fixation protocols, staining variability across laboratories, and inter-observer reliability in morphological assessments require standardization before multi-center validation studies can be meaningful.

## 6. Conclusions

We have proposed a multiparametric decision framework for PTI estimation which, within the limits that were explicitly mentioned in the manuscript, will significantly aid forensic pathologists in properly assessing PTIs.

This approach includes temporal biomarker profiles with systematic assessment of confounding factors such as age, medical history, and circumstances of death, but also differential diagnosis to distinguish traumatic neuroinflammation from hypoxic–ischemic injury, septic encephalopathy, and preexisting neurodegeneration, requiring spatial correlation between inflammatory patterns and identifiable mechanical injury sites. PTI estimates should be expressed as time ranges rather than point values, accompanied by explicit confidence assessments based on biomarker concordance: high confidence requires convergence of at least three independent markers from different biological categories, moderate confidence with two concordant markers, and low confidence with single or discordant markers. All estimates must transparently acknowledge modifying factors and case-specific limitations. This integrated approach, prioritizing the convergence of multiple biological signals over reliance on isolated markers, may provide a more scientifically robust and forensically defensible methodology for post-traumatic interval estimation, reflecting the biological reality that no single biomarker can capture the temporal complexity of the post-traumatic neuroinflammatory cascade across diverse populations and injury contexts.

Translation into routine medicolegal application will require rigorous forensic validation in large, well-characterized human autopsy cohorts, with standardized sampling and post-mortem protocols and integrative multiparametric statistical models (including external validation) to quantify uncertainty and improve PTI estimation.

## Figures and Tables

**Table 1:** Respiratory chain complexes are disrupted after traumatic brain injury.

Cause/Source	Key Molecular Events	Timing and Location	Downstream Consequences
Glutamate excitotoxicity and calcium overload	Massive glutamate release leads to an overactivation of NDMA/AMPA receptors => excessive Calcium influx => activation of NOS, phospholipases, proteases and increased mitochondrial respiration => oveproduction of ROS/RNS [35,36,37,38]	Minutes-hours after injury. Cortex and hippocampus are especially vulnerable.	Mitochondrial depolarization, lipid peroxidation, protein and DNA degradation, DNA oxidation, necrosis, apoptosis, long-term cognitive deficits [35,36,37,38].
Mitochondrial electron transport chain dysfunction	Mechanical or Calcium-related injury to mitochondria => impaired electron transport chain (complexes I–IV), loss of membrane potential, opening of permeability of transition pores => electron leak to O_2_ causing the formation of superoxide => increases the production of ROS (positive feedback) [35,36,39,40,41].	Starts in minutes-hours and persists days-weeks in the pericontusional tissue and the hippocampus	Energy failure (decreased ATP production), activation of cell death pathways, releases of pro-apoptotic factors, propagation of secondary injury [35,36,39,40,41].
NADPH oxidase (NOX1/2/4)	TBI activates (NOX) in neurons, microglia, endothelial cells => transfer of electrons from NADPH to O_2_ => rapid superoxide generation [42,43,44,45].	Before 4 h in blast and impact models, especially in the vascular endothelium and microglia	BBB breakdown via tight junction loss and activation of MMPs; edema; microglial-driven chronic neuroinflammation and neurodegeneration [42,43,44,45].
Nitric oxide synthases (nNOS, iNOS, eNOS)	Calcium dependent nNOS/eNOS and iNOS leads to an increased production of NO, which, in combination with superoxide leads to the formation of peroxynitrite => protein nitration (3-NT) and mitochondrial damage [35,37,46].	nNOS/eNOS—early (minutes-hours); iNOS—hours-days. Present in neurons, glial cells and vessels.	Protein nitration, mitochondrial enzyme inhibition, DNA damage, apoptosis. Was associated with delayed congnitive and behavioral deficits [35,37,46,47].
Neuroinflammation and microglial activation	Microglia and migrated leukocytes release ROS, RNS, citokynes (IL-1β, ΤΝF-α) => positive feedback loop on NOX, NOS and mitochondria [42,46,48,49,50].	Hours–weeks; may persist chronically around the injury and in the white matter	Sustained oxidative stress, synaptic dysfunction, progressive neuronal loss, mood and cognitive disorders [42,46,48,49,50].
BBB disruption and vascular oxidative stress	Oxidative stress in the endothelium => loss of tight junction proteins (claudin-5, occludin, ZO-1), activation of MMP3 and MMP9, degradation of VEGFR-2 => increased permeability of the BBB [44,45,51].	Hours (around 4 h in blast TBI), and may remain abnormal for days.	Vasogenic and cellular edema, infiltration of peripheral immune cells, further ROS/RNS production and neuroinflammation [44,45,51].
Iron and hemoglobin breakdown products	Microhemorrhages and contusion release Fe^2+^, Fe^3+^ and heme => generation of hydroxyl radicals [41,52].	Subacute/chronic, hear hemorrhagic foci	Increased lipid peroxidation, ferroptosis-like death, increased expansion of the injuries [48,51].
Depletion of mitochondrial antioxidants	Decrease in Reduced Glutathione, NADPH, Superoxide Dismutase 2 (SOD2), Peroxiredoxin 2 (PRX2), Thioredoxin 2 (TRX2) in mitochondria, inconstant compensatory increase in Catalase activity => inability to detoxify superoxide and H_2_O_2_ [40,47]	From 30 min to >14 days.	Persistent redox imbalance, increased window of oxidative damage and potential therapeutic activity for antioxidants [47].
Global failure of cytosolic antioxidant systems	Decreased Reduced Glutathione/Oxidized Glutathione ratio, decreased Glutathione Peroxidase, Glutathione Reductase, Glutathione S-Transferase, Glucose-6-Phosphatase Dehydrogenase, SOD, Catalase in brain tissue. Impaired Nuclear Factor Erythroid 2-Related Factor 2 Dehydrogenase (Nrf2-ARE) activation [36,40,53,54].	Duration incompletely mapped in humans.	Decreased capacity to neutralize ROS/RNS, greated susceptibility of lipids, proteins, DNA to oxidation, poor recovery [36,40,53]
Nrf2 pathway supression/insufficient activation	Injury and inflammation supress or inadequately activate Nrf2, liting the induscion of Heme Oxygenase-1, NADPH Quinone Oxidoreductase 1, Gluthathione Peroxidase 1 and other detoxifying enzymes [46,53,54]	Acute and subacute phases. Shown in multiple rodent TBI models [55,56,57]	Exaggerated oxidative damage, apoptosis, neuroinflammation. Nrf2 activators and phytochemicals show neuroprotection preclinically [46,53,54,58]

**Table 2:** Practical summary of key neuroinflammatory biomarkers for approximate post-traumatic interval (PTI) stratification.

Marker/Process	Approximate PTI Bin(s)	Specimen/Readout	Main Limitations/Confounders	Species & Sample Size (Typical)	Experimental/Clinical Model (TBI Type)	Key References
Microglial activation (Iba1/TMEM119/CD68; morphology)	<1 h; 1–6 h; 6–24 h	Brain tissue IHC (cortex/white matter adjacent to lesion)	Many windows derived from rodent models Strong effects of region/severity; PMI and agonal hypoxia can alter morphology	Focal CCI and lateral FPI; diffuse/midline FPI; pediatric/closed-head WD; human lethal TBI with survival <2 h to >3 d	Focal CCI, lateral/midline FPI, repeated/mild rotational models; diffuse TBI in large animals; fatal human TBI with survival <2 h to months	[90,93,102,186,187]
Axonal injury (βAPP; neurofilament changes)	<1 h; 1–6 h; 6–24 h	Brain tissue IHC (white matter tracts, corpus callosum, brainstem)	Overlap with hypoxia/ischemia; Requires pattern-based interpretation and combination with vascular/mechanotransduction markers	Rodents (CCI, weight-drop, FPI, rTBI; ≈5–12/arm); pig (CCI/diffuse; ≈7–10); clinical NfL cohorts (dozens–hundreds of patients)	Diffuse TBI (rotational/weight-drop), focal CCI, rTBI; humans: radiologic DAI, moderate–severe TBI with βAPP post-mortem and blood NfL	[17,22,188,189,190,191,192]
DAMPs (HMGB1 translocation; TLR4/RAGE axis)	1–6 h; 6–24 h	Brain IHC (nuclear → cytoplasmic shift); serum/CSF (context-dependent)	Low specificity (hypoxia-ischemia/systemic inflammation); Influenced by agonal course, resuscitation, sepsis	Mouse/rat ischemia/TBI/SCI (≈6–12/arm); human stroke/TBI cohorts (≈20–100); experimental SCI (≈8–15/arm)	Cerebral ischemia (MCAO), CCI/FPI TBI, SAH, SCI; humans: acute stroke/TBI with plasma/CSF HMGB1, S100B, RAGE	[9,193,194,195,196,197]
Early cytokines (IL-1β, TNF-α)	1–6 h; 6–24 h	Brain tissue, CSF/serum (ELISA/multiplex/IHC)	High inter-individual variability; strongly affected by ventilation/CPR, infection, medications; Temporal windows often preclinical	Rodent CCI/FPI/rTBI (≈6–10/arm, 1–24 h); large mammals (mild diffuse TBI; ≈6–8/arm); human CSF/serum studies (≈20–80)	Focal and diffuse TBI (CCI, FPI, rTBI); ischemia/stroke as analog; ICU TBI patients with serial cytokine monitoring	[90,188,189,198]
Delayed cytokine (IL-6)/chemokine signaling	6–24 h; 1–3 days	CSF/serum and brain tissue (variable)	Non-specific; sustained elevation with systemic inflammation and ICU course	Rodents (CCI/FPI, 6 h–7 d; ≈6–10/arm); clinical stroke/TBI/SAH cohorts (≈30–200)	Acute–subacute focal/diffuse TBI, stroke/hemorrhage; critically ill patients with plasma/CSF IL-6 and chemokines	[83,90,195,198]
Leukocyte infiltration (neutrophils, lymphocytes)	Neutrophils: 1–6 h to 6–24 h; lymphocytes: 1–3 days to >3 days	Brain histology/IHC (perivascular and parenchymal)	Depends on BBB integrity and immune status; altered by hemorrhage, shock, and therapeutic interventions	Mouse/rat CCI/FPI/ischemia/SAH (≈6–12/arm); SCI models (≈8–15/arm); human biopsy/autopsy series (tens of cases)	Sterile acute injuries (focal/diffuse TBI, ischemia, SCI); perivascular/parenchymal histology; linked to HMGB1/RAGE and DAMPs	[194,195,196,199]
BBB disruption (IgG/fibrinogen leakage; TJ proteins; MMP-9)	1–6 h; 6–24 h; 1–3 days	Brain IHC (IgG/fibrinogen; claudin-5/occludin/ZO-1); MMP activity	PMI sensitive; influenced by agonal hypoxia/hypercapnia, ventilation settings, catecholamines	Rodent CCI/FPI/ischemia (≈6–12/arm); pig TBI (≈7–10); stroke/TBI cohorts with plasma MMP-9 (≈30–150)	Focal CCI, FPI/diffuse, ischemia-reperfusion; humans: acute stroke/TBI with imaging plus blood BBB/MMP-9 markers	[193,195,196,198]
Astrocytosis/GFAP	6–24 h; 1–3 days; >3 days	Brain IHC; GFAP in CSF/serum (supportive)	Delayed marker; GFAP also increases in non-traumatic CNS injury; affected by age/comorbidities	Rodent CCI/FPI/rTBI (≈6–12/arm, hours–weeks); pig CCI/diffuse (≈7–10); clinical TBI GFAP cohorts (dozens–hundreds)	Focal CCI, diffuse rotational/rTBI, pediatric/adult TBI; humans: mild–severe TBI with serum/CSF GFAP and imaging correlations	[6,119,179,180]

## Data Availability

No new data were created or analyzed in this study.

## Supplementary Materials

The following supporting information can be downloaded at: https://www.mdpi.com/article/10.3390/ijms27042049/s1.

## Abbreviations

AIS	Abbreviated Injury Scale
AMPA	α-amino-3-hydroxy-5-methyl-4-isoxazolepropionic acid
APOE	Apolipoprotein E
APP	Amyloid Precursor Protein
ASC	Apoptosis-associated Speck-like protein containing a CARD
ATP	Adenosine Triphosphate
BBB	Blood–Brain Barrier
βAPP	Beta-Amyloid Precursor Protein
CCI	Controlled Cortical Impact
CCL2	C-C Motif Chemokine Ligand 2
CCL3	C-C Motif Chemokine Ligand 3
CCL4	C-C Motif Chemokine Ligand 4
CCR2	C-C Chemokine Receptor Type 2
CD3	Cluster of Differentiation 3
CD4	Cluster of Differentiation 4
CD8	Cluster of Differentiation 8
CD11b	Cluster of Differentiation 11b
CD45	Cluster of Differentiation 45
CD206	Cluster of Differentiation 206
cGAS	Cyclic GMP-AMP Synthase
CNS	Central Nervous System
CSF	Cerebrospinal Fluid
CT	Computed Tomography
CTE	Chronic Traumatic Encephalopathy
DAI	Diffuse Axonal Injury
DAMP	Damage-Associated Molecular Pattern
DNA	Deoxyribonucleic Acid
eNOS	Endothelial Nitric Oxide Synthase
ERK	Extracellular Signal-Regulated Kinase
ERK1/2	Extracellular Signal-Regulated Kinase 1/2
FAK	Focal Adhesion Kinase
FPI	Fluid Percussion Injury
GBDPs	GFAP Breakdown Products
GCS	Glasgow Coma Scale
GFAP	Glial Fibrillary Acidic Protein
GPX1	Glutathione Peroxidase 1
GSK-3β	Glycogen Synthase Kinase-3 beta
H2O2	Hydrogen Peroxide
HIF	Hypoxia-Inducible Factor
HMGB1	High-Mobility Group Box 1
HRP	Horseradish Peroxidase
Iba1	Ionized calcium-binding adapter molecule 1
IgG	Immunoglobulin G
IL-1β	Interleukin-1 beta
IL-1R1	Interleukin-1 Receptor Type 1
IL-1ra	Interleukin-1 Receptor Antagonist
IL-4	Interleukin-4
IL-6	Interleukin-6
IL-10	Interleukin-10
IL-15	Interleukin-15
IL-18	Interleukin-18
iNOS	Inducible Nitric Oxide Synthase
ISS	Injury Severity Score
JNK	c-Jun N-terminal Kinase
MAPK	Mitogen-Activated Protein Kinase
MCP-1	Monocyte Chemoattractant Protein-1
MMP	Matrix Metalloproteinase
MMP-1	Matrix Metalloproteinase-1
MMP-2	Matrix Metalloproteinase-2
MMP-7	Matrix Metalloproteinase-7
MMP-9	Matrix Metalloproteinase-9
MPO	Myeloperoxidase
MRI	Magnetic Resonance Imaging
mRNA	Messenger RNA
NADH	Nicotinamide Adenine Dinucleotide (reduced)
NADPH	Nicotinamide Adenine Dinucleotide Phosphate
NCLX	Mitochondrial Na+/Ca2+ Exchanger
NF-68	Neurofilament-68
NF-κB	Nuclear Factor-kappa B
NFL	Neurofilament Light
NLRP3	NLR Family Pyrin Domain Containing 3
NMDA	N-methyl-D-aspartate
nNOS	Neuronal Nitric Oxide Synthase
NO	Nitric Oxide
NOS	Nitric Oxide Synthase
NOX	NADPH Oxidase
NOX1	NADPH Oxidase 1
NOX2	NADPH Oxidase 2
NQO1	NAD(P)H Quinone Dehydrogenase 1
Nrf2	Nuclear Factor Erythroid 2-Related Factor 2
PDE2	Phosphodiesterase 2
Piezo1	Piezo-type Mechanosensitive Ion Channel Component 1
Piezo2	Piezo-type Mechanosensitive Ion Channel Component 2
PMI	Post-Mortem Interval
PRX2	Peroxiredoxin 2
PSD-95	Postsynaptic Density Protein 95
PTI	Post-Traumatic Interval
RAGE	Receptor for Advanced Glycation End-products
RNA	Ribonucleic Acid
RNS	Reactive Nitrogen Species
ROCK	Rho-associated Protein Kinase
ROCK1	Rho-associated Protein Kinase 1
ROS	Reactive Oxygen Species
SAP-97	Synapse-Associated Protein 97
SASP	Senescence-Associated Secretory Phenotype
SNAP-25	Synaptosome-Associated Protein 25
SOD	Superoxide Dismutase
SOD2	Superoxide Dismutase 2
TBI	Traumatic Brain Injury
TGF-β	Transforming Growth Factor beta
TJ	Tight Junction
TLR	Toll-Like Receptor
TLR4	Toll-Like Receptor 4
TMEM119	Transmembrane Protein 119
TNF-α	Tumor Necrosis Factor alpha
TOS	Total Oxidant Status
TRP	Transient Receptor Potential
TRX2	Thioredoxin 2
TSPO	Translocator Protein
TUNEL	Terminal deoxynucleotidyl transferase dUTP nick end labeling
UCH-L1	Ubiquitin Carboxy-terminal Hydrolase L-1
VEGF	Vascular Endothelial Growth Factor
VEGFR-2	Vascular Endothelial Growth Factor Receptor 2
ZO-1	Zonula Occludens-1
