# Supplementary material for: The Dynamics of Neuroinflammation in Traumatic Brain Injury: Molecular Markers Useful for Establishing the Post-Traumatic Interval in Forensic Practice
Source: Int J Mol Sci. 2026 Feb 22;27(4):2049. doi: 10.3390/ijms27042049 (PMC12940811; doi:10.3390/ijms27042049)
Supplement: Supplementary file 1 [file ijms-27-02049-s001.zip › ijms-4096326-supplementary.pdf]

Supplementary files

Table S1. Mechanotransduction and secondary injury after TBI

Component	What it does	Key signalling pathways activated	Main downstream effects	Role in linking primary to secondary injury
Stretch-activated ion channels (e.g., Piezo1/2, TRP) [1–6]	Causes membrane and cytoskeletal stretch during impact or rapid strain	Ca^{2+}- influx activates MAPKs, RhoA/ROCK downstream transcription factors	Neuronal death, myelin protein loss, altered neurogenesis; Leads to TNF-α, IL-1β production; Potential pro-repair signaling	Translate mechanical stretch directly into ionic imbalance and injury signaling within milliseconds of impact
Integrin-mediated mechanosensing[7–10]	Transmits forces through the extracellular matrix-integrin-cytoskeleton complexes Changes the stiffness of the substrate	FAK/Src, MAPKs, NF-κB, cytoskeletal remodeling	Axonal cytoskeletal damage; β-APP accumulation; Modulation of inflammatory and survival pathways	Couples tissue-level strain to axonal skeleton injury May serve as post-mortem marker of traumatic axonal injury
Primary cilium and subcellular structures[7,10,11]	Bends cilium Causes local matrix strain	Ca^{2+}, Wnt, MAPKs, other mechanosensitive cascades	Shifts in cell phenotype; Stress and inflammatory responses; Altered differentiation states	Contribute to "neuropathological mechanotransduction" by sensing micro-scale deformations after TBI
MAPK pathways (ERK, JNK, p38)[1,6,12]	Are activated downstream of Ca^{2+} entry	ERK1/2, JNK, p38	May cause myelin protein loss, apoptosis, or pro-survival responses, depending on the context	Early decision-makers, which may direct cells toward repair vs injury after mechanical insult
NF-κB[13–15]	Triggered by mechanosensitive and redox pathways.	NF-κB nuclear translocation	Induces the production of cytokines, adhesion molecules Amplifies neuroinflammation	Sustains the secondary inflammatory cascades, long after the initial mechanical event
Hypoxia-inducible factors (e.g., HIF-1α) [13,14]	Causes relative hypoxia, perfusion changes, metabolic stress after the impact	HIF-regulated transcription	Increases VEGF production; Causes metabolic reprogramming; May trigger stress responses	Links mechanical-vascular disturbances with longer-term vascular and metabolic pathology

Table S2. Risk factors and their influence on neuroinflammation after TBI

Risk factor	Specific influence on neuroinflammation after TBI	Mechanism	Impact on outcome
Age	Changes the magnitude and duration of microglial and astrocyte activation Aged and very young brains often show exaggerated or prolonged glial responses compared with young adults	Aged brains: primed microglia with higher basal pro-inflammatory cytokines, impaired phagocytosis, increased ROS, senescence markers, and chronic astrogliosis Young brains: stage-specific differences with greater and more persistent microglial activation at certain developmental windows[16–22]	Older animals and patients show larger lesions, more white-matter damage, and worse functional/cognitive outcomes Early-life TBI can also trigger long-lasting glial activation that may influence later vulnerability [16,17,19–22]
Genetic background	Modifies how a given mechanical insult translates into inflammatory and axonal responses; AD-model genotypes can augment or attenuate specific inflammatory readouts	In mild CHIMERA TBI, age and APP/PS1 genotype are known to interact. Older WT mice show increased neuroinflammation after TBI, whereas old APP/PS1 mice have attenuated cytokine/microglial responses despite similar axonal injury; AD-linked pathology and genes (Aβ, tau, APOE) reshape microglial priming and macrophage behavior [22–24] The apolipoprotein E ε4 allele, a well-established risk factor for Alzheimer's disease, associates with poorer outcomes after TBI, potentially through effects on inflammation, lipid metabolism, and amyloid-β accumulation[25]	Genotype can decouple structural injury from inflammatory biomarkers and may alter risk of progressive neurodegeneration or AD-like pathology after TBI[22–24]
Comorbidities. Extracranial injuries	Can modify the systemic–CNS inflammatory crosstalk, changing both the magnitude and "quality" (protective vs maladaptive) of post-traumatic inflammation	Ageing and comorbidities (cardiometabolic disease, chronic inflammatory states) are associated with maladaptive systemic immunity (immunosenescence, chronic low-grade inflammation) and altered recruitment/phenotype of meningeal and brain-infiltrating leukocytes after TBI[17,22,26]	Older, comorbid patients and aged animal models exhibit impaired systemic responses, enhanced meningeal and cortical chemokine expression, more myeloid infiltration, and worse long-term neurological outcomes for comparable TBI severities[17,22,26]

**Pre-existing neuroinflammation. Prior immune challenges Repeated trauma**	"Primed" immune state lay exaggerate or dysregulatedinflammatory cascades to a severity similar to one in which a "non-primed" immune whould show a significantly milder reaction;<br>In some preconditioning contexts, inflammation can be partially protective	Aging, chronic stress, infection, or prior brain injury are known to shift baseline cytokines, ROS handling, and mitochondrial function, and subsequently decreasing the threshold for microglial activation and amplifying responses to TBI;<br>Some controlled pre-injury immune challenges can induce neuroprotective adaptations[22,24,27,28]	Pre-TBI inflammatory burden may be as influential as post-TBI processes in determining chronic pathology and cognitive decline; helps explain heterogeneity of outcomes at similar anatomical severities[22,27,28]

Supplementary references

1. Mocciaro, E.; Kidd, M.; Johnson, K.; Bishop, E.; Johnson, K.; Zeng, Y.P.; Perrotta, C.; Micci, M.-A. Mechanosensitive Ion Channel Piezo1 Modulates the Response of Rat Hippocampus Neural Stem Cells to Rapid Stretch Injury. *PLoS One* **2025**, *20*, e0323191.
2. Beltrán, S.M.; Bobo, J.; Habib, A.; Kodavali, C.V.; Edwards, L.; Mamindla, P.; Taylor, R.E.; LeDuc, P.R.; Zinn, P.O. Author Correction: Characterization of Neural Mechanotransduction Response in Human Traumatic Brain Injury Organoid Model. *Sci. Rep.* **2024**, *14*, 2151.
3. Beltrán, S.M.; Bobo, J.; Habib, A.; Kodavali, C.V.; Edwards, L.; Mamindla, P.; Taylor, R.E.; LeDuc, P.R.; Zinn, P.O. Characterization of Neural Mechanotransduction Response in Human Traumatic Brain Injury Organoid Model. *Sci. Rep.* **2023**, *13*, 13536.
4. Xiao, Y.; Zhang, Y.; Yuan, W.; Wang, C.; Ge, Y.; Huang, T.; Gao, J. Piezo2 Contributes to Traumatic Brain Injury by Activating the RhoA/ROCK1 Pathways. *Mol. Neurobiol.* **2024**, *61*, 7419–7430.
5. Ryu, Y.; Wague, A.; Liu, X.; Feeley, B.T.; Ferguson, A.R.; Morioka, K. Cellular Signaling Pathways in the Nervous System Activated by Various Mechanical and Electromagnetic Stimuli. *Front. Mol. Neurosci.* **2024**, *17*, 1427070.
6. Kim, J.; Adams, A.A.; Gokina, P.; Zambrano, B.; Jayakumaran, J.; Dobrowolski, R.; Maurel, P.; Pfister, B.J.; Kim, H.A. Mechanical Stretch Induces Myelin Protein Loss in Oligodendrocytes by Activating Erk1/2 in a Calcium-Dependent Manner. *Glia* **2020**, *68*, 2070–2085.
7. Keating, C.E.; Cullen, D.K. Mechanosensation in Traumatic Brain Injury. *Neurobiol. Dis.* **2021**, *148*, 105210.
8. Yijie, D.; Weisheng, H.; Ji, Z.; Jiao, M.; Yiwu, Z.; Hongmei, D. Role of Integrin and Its Potential as a Novel Postmortem Biomarker in Traumatic Axonal Injury. *Int. J. Legal Med.* **2023**, *137*, 843–849.
9. Stukel, J.M.; Willits, R.K. Mechanotransduction of Neural Cells through Cell-Substrate Interactions. *Tissue Eng. Part B Rev.* **2016**, *22*, 173–182.
10. Miles, L.; Powell, J.; Kozak, C.; Song, Y. Mechanosensitive Ion Channels, Axonal Growth, and Regeneration. *Neuroscientist* **2023**, *29*, 421–444.
11. Nikolaev, Y.; Cox, C.D.; Ridone, P.; Rohde, P.R.; Cordero-Morales, J.F.; Vasquez, V.; Laver, D.R.; Martinac, B. Mammalian TRP Ion Channels Are Insensitive to Membrane Stretch. *Biophys. J.* **2020**, *118*, 22a.

12. Cheng, P.-W.; Wu, Y.-C.; Wong, T.-Y.; Sun, G.-C.; Tseng, C.-J. Mechanical Stretching-Induced Traumatic Brain Injury Is Mediated by the Formation of GSK-3β-Tau Complex to Impair Insulin Signaling Transduction. *Biomedicines* **2021**, *9*, 1650.
13. Bhowmick, S.; D'Mello, V.; Caruso, D.; Muneer, P.M.A. Correction to: Traumatic Brain Injury-Induced Downregulation of Nrf2 Activates Inflammatory Response and Apoptotic Cell Death. *J. Mol. Med. (Berl.)* **2025**, *103*, 489.
14. Jarrahi, A.; Braun, M.; Ahluwalia, M.; Gupta, R.V.; Wilson, M.; Munie, S.; Ahluwalia, P.; Vender, J.R.; Vale, F.L.; Dhandapani, K.M.; et al. Revisiting Traumatic Brain Injury: From Molecular Mechanisms to Therapeutic Interventions. *Biomedicines* **2020**, *8*, 389.
15. Wachtler, N.; O'Brien, R.; Ehrlich, B.E.; McGuone, D. Exploring Calcium Channels as Potential Therapeutic Targets in Blast Traumatic Brain Injury. *Pharmaceuticals (Basel)* **2025**, *18*, 223.
16. Delage, C.; Taib, T.; Mamma, C.; Lerouet, D.; Besson, V.C. Traumatic Brain Injury: An Age-Dependent View of Post-Traumatic Neuroinflammation and Its Treatment. *Pharmaceutics* **2021**, *13*, 1624.
17. Ritzel, R.M.; Doran, S.J.; Glaser, E.P.; Meadows, V.E.; Faden, A.I.; Stoica, B.A.; Loane, D.J. Old Age Increases Microglial Senescence, Exacerbates Secondary Neuroinflammation, and Worsens Neurological Outcomes after Acute Traumatic Brain Injury in Mice. *Neurobiol. Aging* **2019**, *77*, 194–206.
18. Wangler, L.M.; Godbout, J.P. Microglia Moonlighting after Traumatic Brain Injury: Aging and Interferons Influence Chronic Microglia Reactivity. *Trends Neurosci.* **2023**, *46*, 926–940.
19. Moro, F.; Pischiutta, F.; Portet, A.; Needham, E.J.; Norton, E.J.; Ferdinand, J.R.; Vegliante, G.; Sammali, E.; Pascente, R.; Caruso, E.; et al. Ageing Is Associated with Maladaptive Immune Response and Worse Outcome after Traumatic Brain Injury. *Brain Commun.* **2022**, *4*, fcac036.
20. Kumar, A.; Stoica, B.A.; Sabirzhanov, B.; Burns, M.P.; Faden, A.I.; Loane, D.J. Traumatic Brain Injury in Aged Animals Increases Lesion Size and Chronically Alters Microglial/Macrophage Classical and Alternative Activation States. *Neurobiol. Aging* **2013**, *34*, 1397–1411.
21. Green, T.R.F.; Murphy, S.M.; Ortiz, J.B.; Rowe, R.K. Age-at-Injury Influences the Glial Response to Traumatic Brain Injury in the Cortex of Male Juvenile Rats. *Front. Neurol.* **2022**, *12*, 804139.
22. Houle, S.; Kokiko-Cochran, O.N. A Levee to the Flood: Pre-Injury Neuroinflammation and Immune Stress Influence Traumatic Brain Injury Outcome. *Front. Aging Neurosci.* **2022**, *13*, 788055.
23. Cheng, W.H.; Stukas, S.; Martens, K.M.; Namjoshi, D.R.; Button, E.B.; Wilkinson, A.; Bashir, A.; Robert, J.; Cripton, P.A.; Wellington, C.L. Age at Injury and Genotype Modify Acute Inflammatory and Neurofilament-Light Responses to Mild CHIMERA Traumatic Brain Injury in Wild-Type and APP/PS1 Mice. *Exp. Neurol.* **2018**, *301*, 26–38.
24. Kokiko-Cochran, O.N.; Godbout, J.P. The Inflammatory Continuum of Traumatic Brain Injury and Alzheimer's Disease. *Front. Immunol.* **2018**, *9*, 672.
25. Mahley, R.W.; Huang, Y. Apolipoprotein e Sets the Stage: Response to Injury Triggers Neuropathology. *Neuron* **2012**, *76*, 871–885.
26. Barrett, J.P.; Knoblach, S.M.; Bhattacharya, S.; Gordish-Dressman, H.; Stoica, B.A.; Loane, D.J. Traumatic Brain Injury Induces CGAS Activation and Type I Interferon Signaling in Aged Mice. *Front. Immunol.* **2021**, *12*, 710608.

27. Norden, D.M.; Muccigrosso, M.M.; Godbout, J.P. Microglial Priming and Enhanced Reactivity to Secondary Insult in Aging, and Traumatic CNS Injury, and Neurodegenerative Disease. *Neuropharmacology* **2015**, *96*, 29–41.
28. Bray, C.E.; Witcher, K.G.; Adekunle-Adegbite, D.; Ouvina, M.; Witzel, M.; Hans, E.; Tapp, Z.M.; Packer, J.; Goodman, E.; Zhao, F.; et al. Chronic Cortical Inflammation, Cognitive Impairment, and Immune Reactivity Associated with Diffuse Brain Injury Are Ameliorated by Forced Turnover of Microglia. *J. Neurosci.* **2022**, *42*, 4215–4228.
